# Canonical Discrimination of the Effect of a New Broiler Production Facility on Soil Chemical Profiles as Related to Current Management Practices

**DOI:** 10.1371/journal.pone.0128179

**Published:** 2015-06-01

**Authors:** Cynthia L. Sheffield, Tawni L. Crippen, J. Allen Byrd, Ross C. Beier, Kathleen Yeater

**Affiliations:** 1 Food and Feed Safety Research Unit, Southern Plains Agricultural Research Center, Agricultural Research Service, United States Department of Agriculture, College Station, Texas, United States of America; 2 Plains Area, Agricultural Research Service, United States Department of Agriculture, Fort Collins, Colorado, United States of America; Catalan Institute for Water Research (ICRA), SPAIN

## Abstract

The effect dirt-floored broiler houses have on the underlying native soil, and the potential for contamination of the ground water by leaching under the foundation, is an understudied area. This study examines alterations in fifteen quantitative soil parameters (Ca, Cu, electrical conductivity, Fe, K, Mg, Mn, Na, NO_3_, organic matter, P, pH, S, soil moisture and Zn) in the underlayment of a newly constructed dirt-floored broiler house over the first two years of production (Native through Flock 11). The experiment was conducted near NW Robertson County, Texas, where the native soil is a fine, smectitic thermic Udertic Paleustalfs and the slopes range from zero to three percent. Multiple samples were collected from under each of three water and three feed lines the length of the house, in a longitudinal study during February 2008 through August 2010. To better define the relationship between the soil parameters and sampling times, a canonical discriminant analysis approach was used. The soil profiles assembled into five distinctive clusters corresponding to time and management practices. Results of this work revealed that the majority of parameters increased over time. The management practices of partial and total house clean-outs markedly altered soil profiles the house underlayment, thus reducing the risk of infiltration into the ground water near the farm. This is important as most broiler farms consist of several houses within a small area, so the cumulative ecological impact could be substantial if not properly managed.

## Introduction

Approximately 40–50% of the Earth’s terrestrial surface is currently used for agriculture [1]; this is likely to increase as a result of increased human populations and their associated demand for agricultural products [2]. Agricultural non-source contamination of surface is viewed by the U.S. Environmental Protection Agency as a major threat to various water and riparian ecosystems [3]. The increasing demand for animal products around the world has resulted in increased numbers of poultry production facilities. Global poultry meat exports are projected to exceed 10 million metric tons by 2020 [4]. The poultry industry is responding to this challenge by increasing the capacity and productivity of production operations. In the United States, currently eight states (Alabama, Arkansas, Georgia, Kentucky, Mississippi, North Carolina, and Texas) deliver 78% of U.S. broiler production [5]. Geographical concentration of the broiler industry is closely coupled with feed/nutrient import and positive nutrient imbalance on agricultural land [6].

One of the paramount challenges of the twenty-first century will be meeting society’s increasing food needs while controlling pathogens and at the same time reducing the negative environmental impacts of agriculture. Environmental sustainability cannot be achieved without the maintenance of soil quality. Many soil factors interact with each other, and thus, the value of one is affected by one or more other factors. The critical limits of selected soil factors can be defined as the advantageous range of values that must be maintained for the healthy functioning of the soil ecosystem [7]. One of America’s most extensive, costly and challenging environmental problems is the nutrient pollution of surface and ground water. Since the mid to late 1990’s it has been well documented that the application of animal waste often results in the pollution of surface and ground water [8]. The primary source of animal production related nutrient pollution is the runoff of animal manures. In the Mississippi River Basin, which encompasses 31 states and finally drains into the Gulf of Mexico, nutrients from animal feeding operations contribute the most to this pollution. Nutrient pollution damages the environment, especially water quality. The process of eutrophication (hypertrophication) of surface waters is the ecosystem response to the addition of nutrients (especially phosphorus) to an aquatic system. Eutrophication is costly to the environment and society. According to the EPA, problems with nutrient pollution have far-reaching impacts on the U.S. economy impacting many sectors that depend on clean water. The tourism industry alone loses about $1 billion dollars yearly due to the losses in recreational activities, such as fishing and outdoor aquatic sports. Further, nutrient pollution causes annual losses to the commercial fishing and shellfish industries reaching into the tens of millions of dollars each year. Finally, recovery from eutrophication has been estimated to take a thousand or more years, as the damage is not isolated to the aquatic ecosystem, but impacts all other ecosystems dependent on these aquatic systems.[9—10]After an extensive search of the literature, these authors could find no studies which evaluated the potential effects that dirt-floored broiler houses have on the underlying native soil and the health of the surrounding environment as poultry production progressed. Some researchers in the past have examined poultry houses after they had been in continuous use for 10 to 45 years; however even these studies only evaluated the levels of nitrogen [11] or nitrogen and phosphorus [12]. Kratz et al. [13] examined free-range and organic broiler operations evaluating both nitrogen and phosphorus. This study follows the alterations in soil parameters from a native environment, prior to placement of a commercial dirt-floored broiler production facility, through several flock rotations. Additionally, no previous studies incorporated the effects of management practices (clean-outs) on the soil parameters. The objective of this study was to examine alterations in fifteen physiochemical parameters: Ca, Cu, electrical conductivity (EC), Fe, K, Mg, Mn, Na, NO_3_, organic matter (OM), P, pH, S, soil moisture (SM) and Zn; in the soil prior to and following the placement of a newly constructed commercial dirt-floored broiler house as poultry production progressed over 2.5 years. This time period corresponded to 11 flock rotations.

## Materials

### Site Description

Several new dirt-floored broiler production houses were constructed on an open range greater than 1 mile from the nearest pre-existing broiler production facility in NW Robertson County, Texas. Permits and approvals for the work were not needed, as the land was privately owned and we had permission to conduct this research from the land owner. The soil was a fine, smectitic thermic Udertic Paleustalfs with slopes ranging from zero to three percent [14]. The research was conducted from February 2008 to August 2010. Twenty five centimeters of commercial grade clay-based topsoil was used for the house foundation pad. The broiler facility was a standard tunnel ventilated metal house 14 m wide and 152.4 m long. Alternating water and feed lines ran the entire length of the house spaced at 1.52, 2.44, 3.66, 4.57 and 6.10 m from the North and South walls. Over the research period 11 flocks were grown out in the facility, over an average duration of 59 ± 6 d.

### Management Practices

Each flock contained 25,800 birds, placed in the house at 1 d of age and confined to half of the house for 2 wk, then allowed access to the entire house. After the 7^th^ flock rotation, the producer performed a partial clean-out. This consisted of removal of the caked top layer of hardened manure along with 5.08–7.62 cm of litter. Fresh bedding of pine chips (5.08–7.62 cm) was then added to the house. A total clean-out was performed after the 9^th^ flock rotation. This involved removal of all litter plus 1.27–2.54 cm of the pad-soil. Fresh pine chip bedding (15.24 cm) was then added to the house.

### Sample Collection

Prior to ground breaking for the new construction, the location of the house was plotted and the top 7.62 cm of native soil was randomly sampled n = 27 the length and width of the proposed house and then pooled (9 each) into 3 samples. After addition of 25.4 cm of pad soil to the site, n = 27 random samples of the top 7.62 cm of pad soil were collected and pooled (9 each) into 3 samples for analysis. After construction of the poultry house, approximately 15.24 cm of pine chip bedding was placed throughout the house. After placement of bedding, the top 7.62 cm of pad soil was collected by removing the litter to access the soil beneath. For Flocks 1, 2 and 4, soil was collected five times (on alternate weeks) during the grow-out period. For all other flocks soil was collected at the beginning within 1 d after bird placement and at the end of the grow-out period within 1 d after bird removal. Composite samples were made from samples collected every two weeks of the flock rotation, for Flocks 1 and 2 (n = 60). Due to logistical limitations in sampling times with the farm producer, flock 4 had an n = 48 and flocks 3, 5–11 had an n = 24 samples. Soil was collected using a five inch long spade, which was thoroughly cleaned with Clorox wipes between sample sites; each sample was placed into individual sterile zip-top bags. For collection purposes, the house was divided into Side A and Side B. For the first 2 wks of each flock rotation the birds were restricted to Side B by a brooder fence. Three replicate samples were collected from each side at random intervals along each of 3 feed and 3 water lines (n = 36) for each time point. The 4^th^ water line was not sampled. The total number of soil samples collected over the course of the study was 1116. The 3 replicate samples for each side and line for each time point were combined (n = 12) and the composite sample used for soil analysis (n = 372). Analysis was performed at Texas A&M AgriLife Extension Soil, Water and Forage Testing Laboratory (College station, TX). All samples were oven dried at 65°C (± 2°C) in a forced air oven for 16 hours or until dry, then pulverized using an open mesh bottom hammer style soil pulverizer (Humboldt Mfg. Co., Schiller Park, IL). The sample was filtered to remove particles >2mm. A 1:2 soil: water extract using deionized water was made. Samples were stirred and allowed to equilibrate for 30 minutes and the pH determined using a hydrogen selective electrode [15]. Electrical conductivity (μScm^-1^) was also determined using a conductivity probe [16]. The percentage of soil moisture was determined using a gravimetric method. The percentage of organic matter was determined using the method described by Wang and Anderson [17] and modified by using an Eltra Helios CN analyzer (ELTRA-Africa, Selcourt, South Africa) running at 675°C.

Nitrate-nitrogen (NO_3_-N) was extracted from soils using a 1 N KCl solution. Nitrate was determined by reduction of nitrate (NO_3_-N) to nitrite (NO_2_-N) using a cadmium column followed by spectrophotometric measurement [18—19]. The content of P, K, Ca, Mg, Na and S were extracted using a Mehlich III solution (GFS Chemicals, Inc., Powell, OH) and determined by Inductively Coupled Argon Plasma Optical Emission Spectrophotometry (ICP) [20—21]. Micronutrients Cu, Fe, Mn and Zn were extracted using a 0.005 M diethylene-triamine-pentaacetic acid (DTPA), 0.01 M CaCl_2_ and 0.10 M triethanolamine solution. A minimum of 20 g of soil was either volumetrically or gravimetrically placed in an extraction cup with 40 ml DTPA solution; agitated at 180 rpm for 120 min prior to filtration through a grade-2 filter paper (Whatman). The analytes were determined by ICP [22].

### Statistical Analysis

Initially analyses were performed by ANOVA with Bonferroni’s multiple comparison post-test using GraphPad Prism version 5.00 (GraphPad Software, San Diego California USA). Results demonstrated no significant differences between water versus feed line collection sites, or Side A versus Side B collection sites. Therefore, these samples were combined based on flock rotation for further analysis. The soil parameters of the native, pad, and flock soil samples were analyzed using a canonical discriminant analysis (CDA) approach. All 15 parameters were included as variables and the 13 data collection groups were defined as the class levels for the analysis. These analyses were performed using JMP (version 11.2; SAS Institute, Inc., Cary, NC) with the discriminant analysis procedure. Additionally, Mahalanobis distance, D^2^, between classes was calculated using SAS (version 9.4; SAS Institute, Inc., Cary, NC) CANDISC procedure with the distance option. For all analyses reported in this work any p-value ≤ 0.05 was considered significant Table 1.

**Table 1:** Standardized canonical loading coefficients of the first two canonical variates for the 15 soil parameters.

Parameters	Canonical Variates	
	1	2
Electrical Conductivity, uScm^-1^	0.3494	-0.1378
Fe, ppm	-0.1423	-0.0569
NO_3_, ppm	-0.1804	-0.1181
Soil Moisture, %	-0.0382	0.2575
pH	**0.5239**	0.2292
Ca, ppm	-0.3938	0.0914
K, ppm	0.4637*	-0.2539
Mg, ppm	**0.8254**	**-0.8570**
Na, ppm	-0.5167	0.0735
P, ppm	-0.4406	0.0895
S, ppm	**0.4765**	-0.1734
Cu, ppm	-0.1728	**-0.4543**
Mn, ppm	-0.1382	**0.5195**
Zn, ppm	0.2256	**1.909**
Organic Matter, %	0.3483	**-0.6493**
Canonical correlation	0.9644	0.9372
*P* level of significance	<0.0001	<0.0001
Variance accounted for, %	48.41	26.26

* The parameters with the largest influence for each canonical variate are in bold.

## Results and Discussion

**Fig 1:**
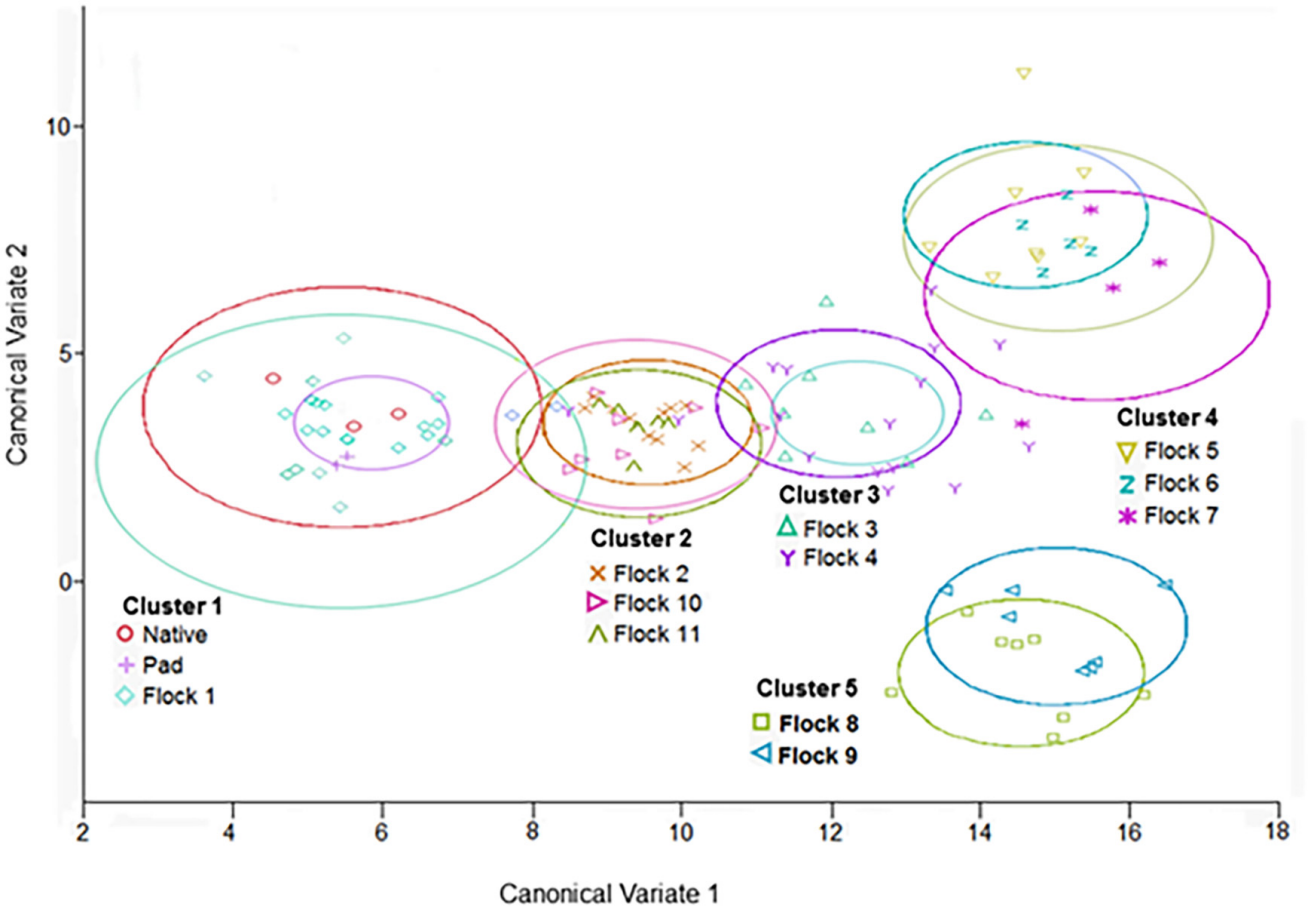
Canonical discriminant analysis plot of clusters from eleven flock rotations. Canonical discriminant analysis plot showing the eleven flocks separated into five discreet clusters (Cluster 1 = Native, Pad and Flock1; Cluster2 = Flock 2, 10 and 11; Cluster 3 = Flocks 3 and 4; Cluster 4 = Flocks 5, 6, and 7; Cluster 5 = Flocks 8 and 9) with the shifts between the clusters along both the Canonical Variate 1 and 2 axes.

The eleven flock rotations separated into five discreet clusters (C1 = Native, Pad and Flock1; C2 = Flock 2, 10 and 11; C3 = Flocks 3 and 4; C4 = Flocks 5, 6, and 7; C5 = Flocks 8 and 9) Table 2 with specific parameters driving the shifts between the clusters (Fig 1). This discretization of the sampling flocks was verified with the Mahalanobis Distance Criterion, *D* ^*2*^, which measured the separation of the clusters, and all pairwise distances between the centroids (cluster means) were significant (*P<*0.0001).

**Table 2:** Sample classes in each canonical discrimination cluster.

Cluster 1	Cluster 2	Cluster 3	Cluster 4	Cluster 5
Native, Pad Flock 1	Flock 2, Flock10, Flock 11	Flock 3, Flock 4	Flock 5, Flock 6, Flock 7	Flock 8, Flock 9

### Interpretation of Canonical Discriminant Analysis

The soil parameters measured were used to discriminate among the flocks and native and pad environments. The first two canonical variates accounted for ~77% of the among-classes variance Table 1. Each canonical variate is the linear combination of the independently measured soil parameters and is orthogonal to the others [23]. The significant (*P<*0.0001) canonical correlations between the flock classes and the first and second canonical variates (*r*_c1_ = 0.96 and *r*_c2_ = 0.94) indicate that the canonical variates explain the differentiation of the flocks and native and pad soil environments.

Canonical loadings measure the simple linear correlation between the original independent soil parameter and the canonical variate, and are interpreted as the relative contribution of each variable to each canonical variate function [24—26]. The first canonical function is dominated by large positive loadings from Mg, S, K, pH, and negative loadings for Na and P Table 1. The second canonical function is dominated by a large positive loading from Zn, followed by a negative Mg loading.

### Parameters (Fe, NO_3,_ EC, and SM)

**Fig 2:**
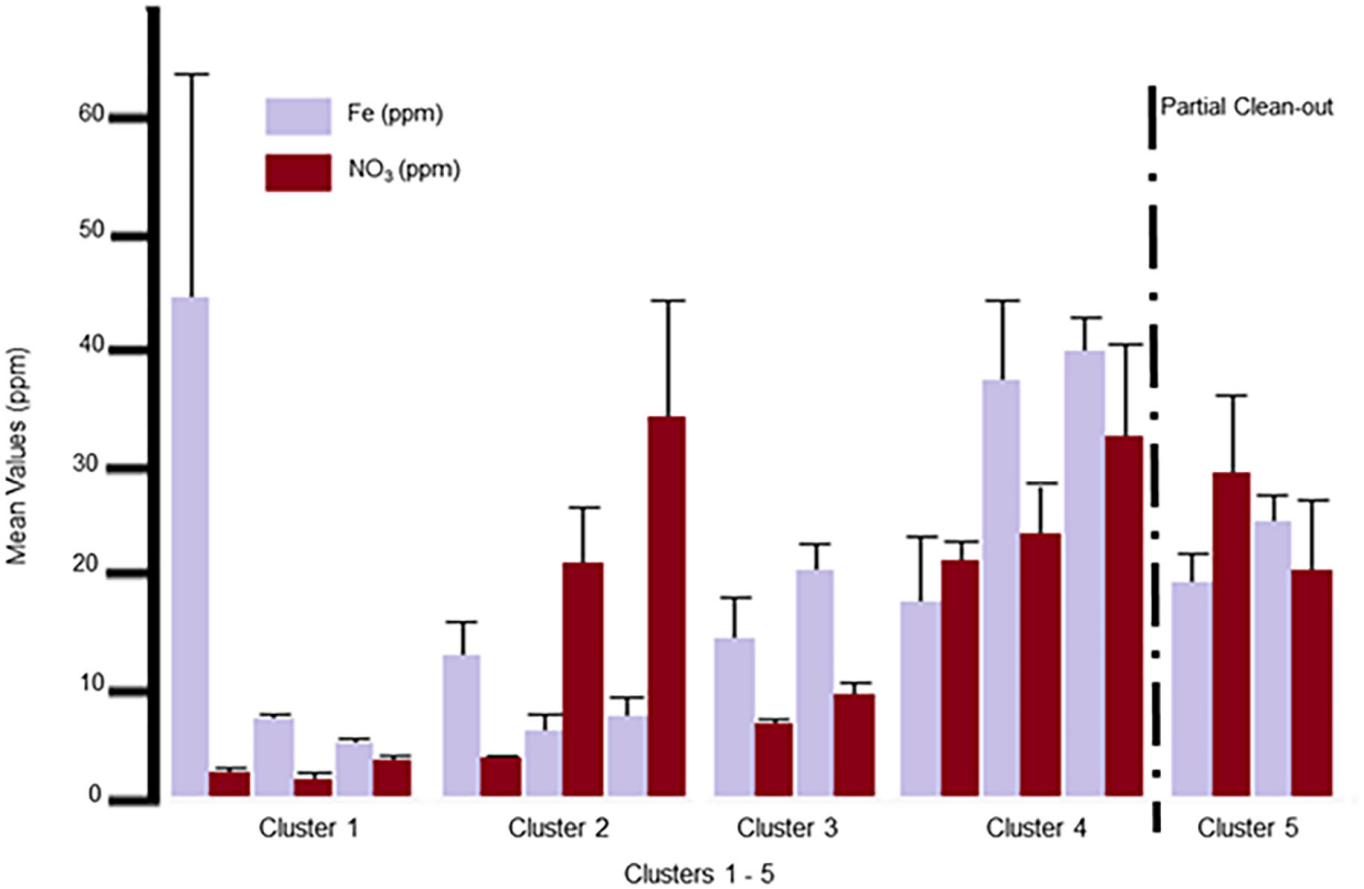
Mean values for the parameters Fe and NO_3_. Mean values for the parameters Fe and NO_**3**_ expressed as ppm, with standard deviation error bars are shown in this graph. In order from left to right on the graph is Cluster 1: Native, Pad and Flock1; Cluster 2: Flock 2, 10 and 11; Cluster 3: Flocks 3 and 4; Cluster 4: Flocks 5, 6, and 7; and Cluster 5: Flocks 8 and 9. The partial clean-out which occurred between Flock 7 and 8 is marked by a dashed line.

**Fig 3:**
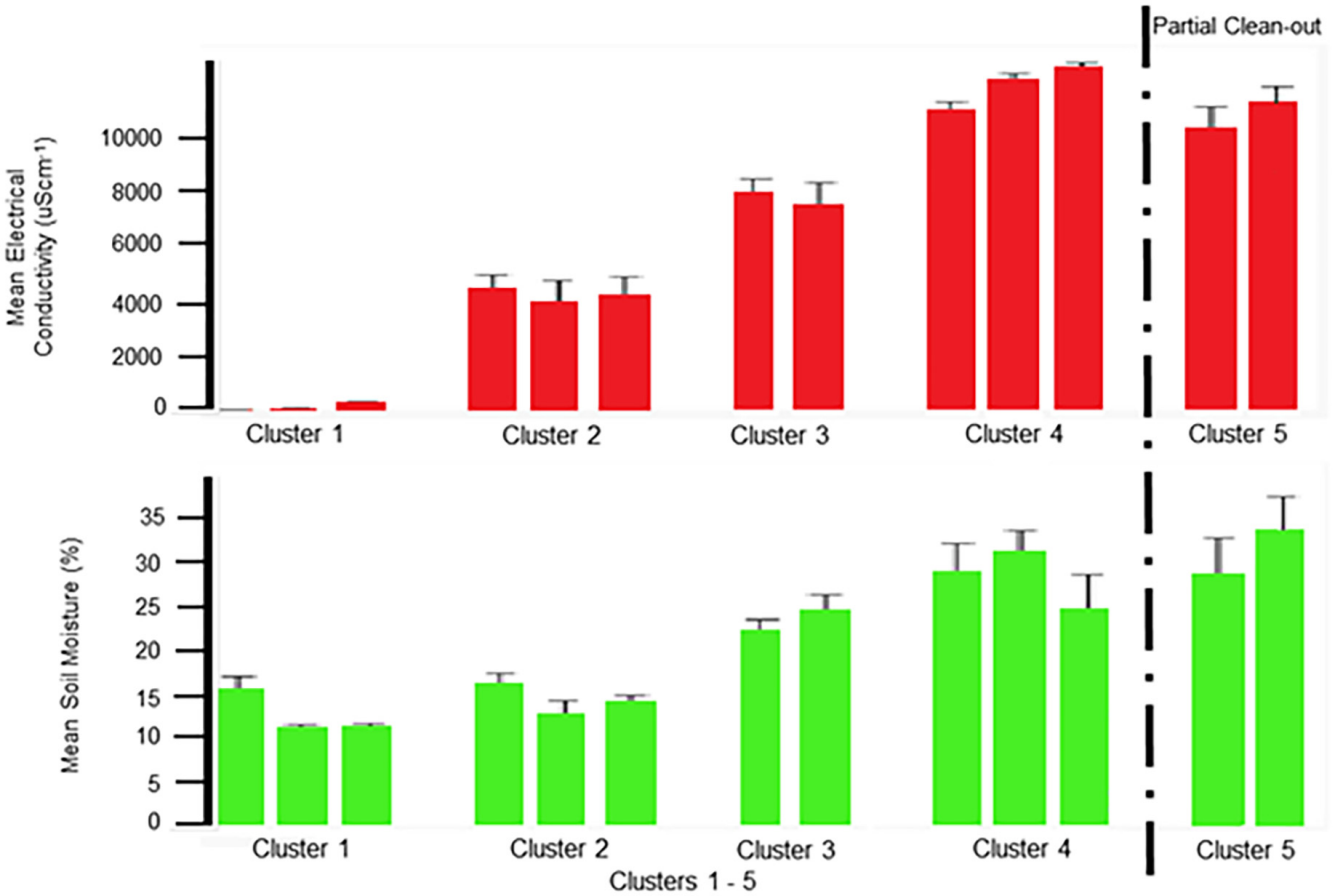
Mean values for Electrical Conductivity. Mean values for Electrical Conductivity, expressed as μScm^-1^, with standard deviation error bars are shown in the top graph. Mean values for Soil Moisture, expressed as a percentage, with standard deviation error bars are shown in the bottom graph. In order from left to right on the graph is Cluster 1: Native, Pad and Flock1; Cluster 2: Flock 2, 10 and 11; Cluster 3: Flocks 3 and 4; Cluster 4: Flocks 5, 6, and 7; and Cluster 5: Flocks 8 and 9. The partial clean-out which occurred between Flock 7 and 8 is marked by a dashed line.

The parameters (Fe, NO_3,_ EC, and SM) Table 1 did not have any significant correlation to the remaining parameters. However, these parameters showed significant increases as successive flocks were reared within the poultry facility. The Fe and NO_3_ were reduced by both the partial and total clean-outs (Fig 2). While no significant effect was observed for EC or SM (Fig 3) following the partial clean-out; both were reduced following the total clean-out. None of these parameters plateaued prior to the clean-out procedures, so it is not possible to predict if increases would continue with successive flocks or when a peak value might be reached.

### Parameter (pH)

**Fig 4:**
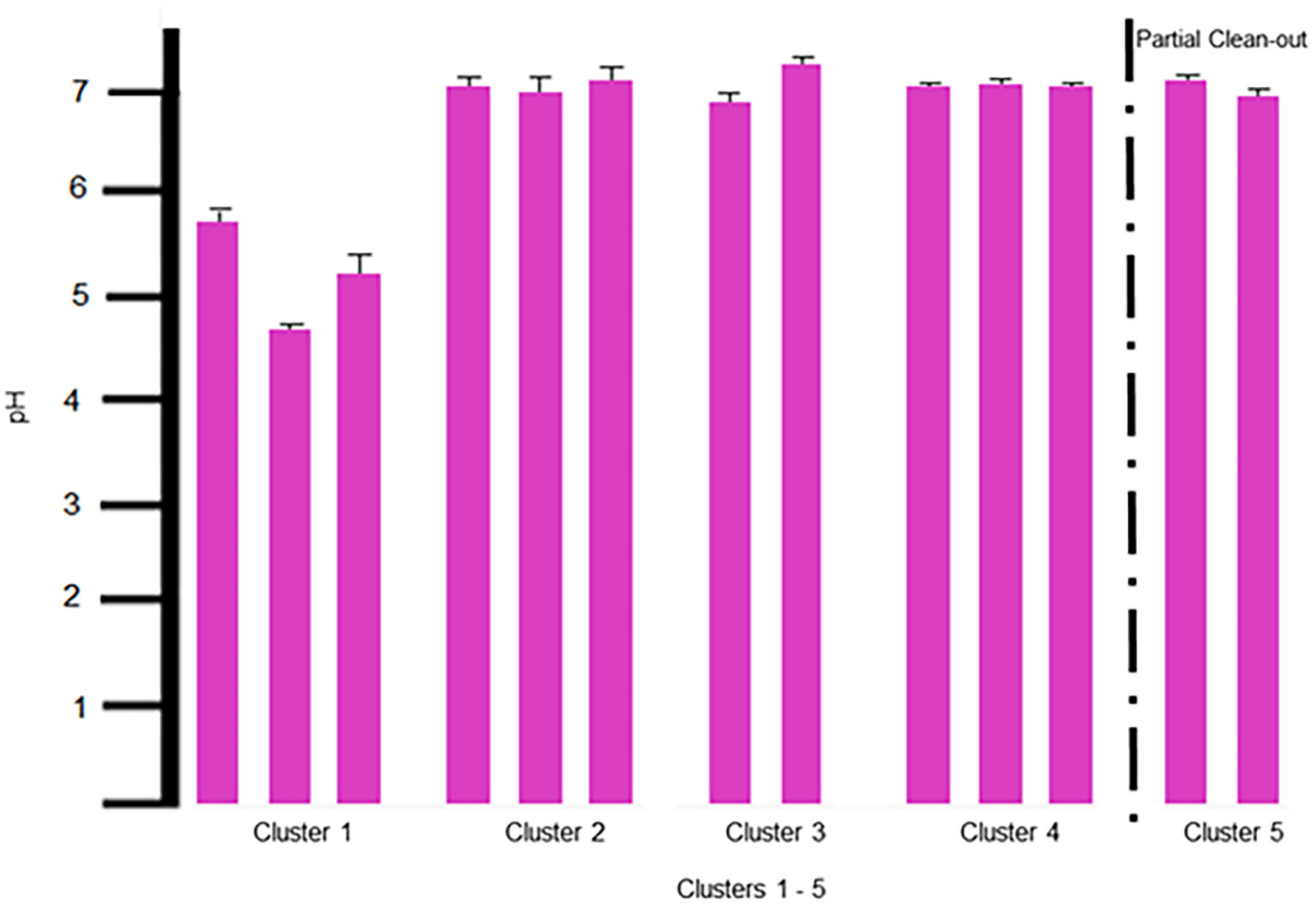
Mean values for soil pH. Mean values for soil pH with standard deviation error bars are shown in this graph. In order from left to right on the graph is Cluster 1: Native, Pad and Flock1; Cluster 2: Flock 2, 10 and 11; Cluster 3: Flocks 3 and 4; Cluster 4: Flocks 5, 6, and 7; and Cluster 5: Flocks 8 and 9. The partial clean-out which occurred between Flock 7 and 8 is marked by a dashed line.

Unlike the other parameters in this group, soils pH (4.95 to 6.40) was significantly more acidic in C1 than that observed for any other cluster (Fig 4). The soil pH in C2–5 were slightly alkaline (7.30 to 7.69) and appeared to stabilize with no additional significant changes [27]. To the broiler producer, the slightly alkaline pH values observed in the soil from C2–5 are of concern because of an increased risk of ammonia volatilization [28], which could lead to potential health problems within the broiler house. Neither the partial or total clean-out significantly affected the slightly alkaline soil pH in subsequent flocks.

### Parameters (Ca, K, Mg, Na, P, and S)

**Fig 5:**
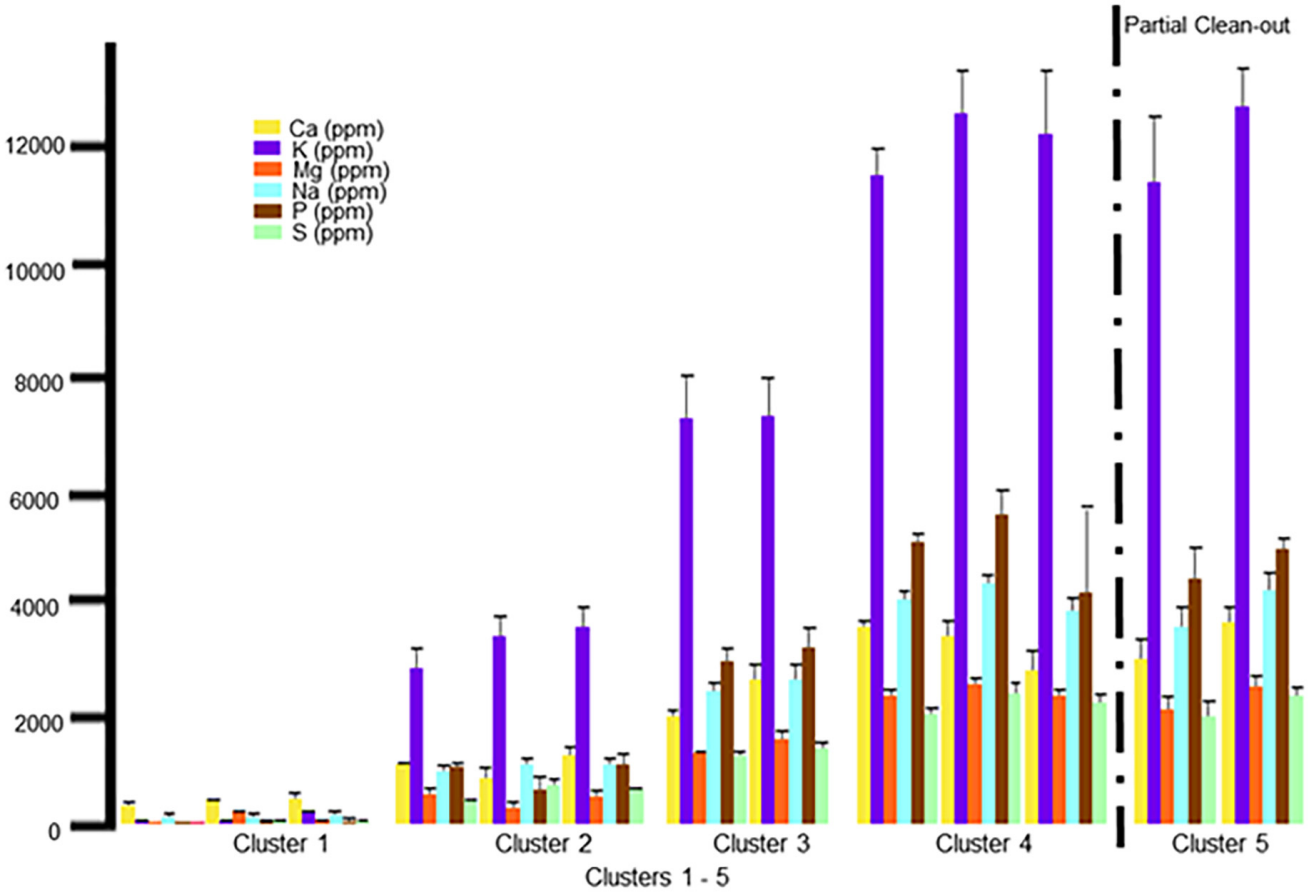
Mean values for the parameters Ca, K, Mg, Na, P and S. Mean values for the parameters Ca, K, Mg, Na, P and S, expressed as ppm, with standard deviation error bars are seen in this graph. In order from left to right on the graph is Cluster 1: Native, Pad and Flock1; Cluster 2: Flock 2, 10 and 11; Cluster 3: Flocks 3 and 4; Cluster 4: Flocks 5, 6, and 7; and Cluster 5: Flocks 8 and 9. The partial clean-out which occurred between Flock 7 and 8 is marked by a dashed line.

The soil parameters (Ca, K, Mg, Na, P, and S) demonstrated the most changes over time and in relationship with the total house clean-out (Fig 5). The values for these parameters were not different within C1; this is reasonable as the manure and urine deposited by the birds during the first flock rotation would likely be absorbed by the 15.24 cm layer of bedding and had not yet influenced the composition of the underlying soil. Significant increases in these parameters were observed over the successive flock rotations, reaching maximum levels within C4. The partial clean-out, conducted after Flock 7, produced no significant reduction in any of these parameters. However, a significant reduction in all of these parameters was achieved with the total house clean-out procedure. In fact, after this management procedure each of these factors returned to levels not significantly different from those observed in Flock 2. While not succeeding in reducing the level back to the pre-flock values of the native or pad soils, this clearly demonstrates that total clean-outs can substantially reduce the risk for infiltration of these parameters into the soil underlayment and possibly the ground water and the potential associated pollution Table 3.

**Table 3:** Key pollutants of environmental interest from poultry manure.

Pollutant	Pathways to the Environment	Potential Impacts
Calcium	• Overland discharge	• Reduction in aquatic life
	• Leachate into ground water	• Increased drinking water treatment cost
Copper	• Overland discharge	• Aquatic toxicity at elevated concentrations
	• Leachate into ground water	
Magnesium	• Overland discharge	• Reduction in aquatic life
	• Leachate into ground water	• Increased drinking water treatment cost
Manganese	• Overland discharge	• Aquatic toxicity at elevated concentrations
	• Leachate into ground water	
Phosphorus	• Overland discharge	• Eutrophication and HABs
	• Leachate into ground water (water soluble forms)	
Potassium	• Overland discharge	• Increased salinity in surface and ground water
	• Leachate into ground water	
Sodium	• Overland discharge	• Reduction in aquatic life
	• Leachate into ground water	• Increased soil salinity
		• Increased drinking water treatment cost
Sulfur (sulfate)	• Overland discharge	• Reduction in aquatic life
	• Leachate into ground water	• Increased drinking water treatment cost
Zinc	• Overland discharge	• Aquatic toxicity at elevated concentrations
	• Leachate into ground water	

### Parameters (Cu, Mn, and Zn)

**Fig 6:**
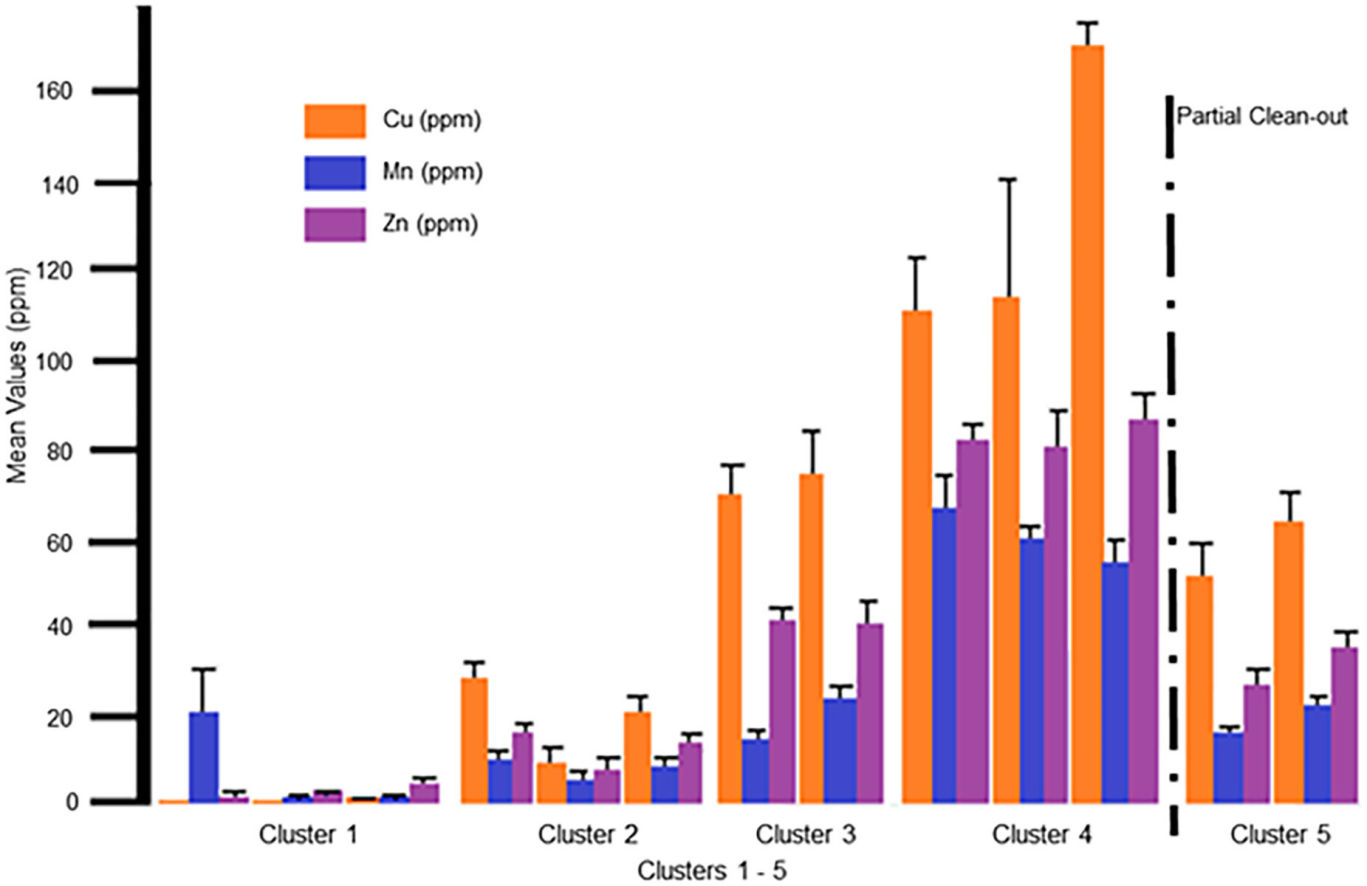
Mean values for the parameters Cu, Mn and Zn Mean values for the parameters Cu, Mn and Zn, expressed as ppm, with standard deviation error bars are seen in this graph. In order from left to right on the graph is Cluster 1: Native, Pad and Flock1; Cluster 2: Flock 2, 10 and 11; Cluster 3: Flocks 3 and 4; Cluster 4: Flocks 5, 6, and 7; and Cluster 5: Flocks 8 and 9. The partial clean-out which occurred between Flock 7 and 8 is marked by a dashed line.

These parameters are considered micronutrients and heavy metals (Fig 6). The levels of these parameters significantly increased over successive flock rotations, with the largest increase (26-fold) occurring between Flock 1 (when birds were initially introduced) and Flock 2. With each successive flock rotation cluster prior to the partial clean-out after Flock 7, these parameters continued to increase three-fold from C2 to C3 and two-fold from C3 to C4. These parameters were reduced two-fold by the partial clean-out and were the driving elements causing the negative shift along the second canonical variate that is observed from C4 to C5 (Fig 1). The specific process that leads to this discrimination is not clear, as no soil (only litter) is removed during the partial clean-out. This requires further investigation. The total clean-out procedure, did incorporate the removal of soil (1.27–2.54 cm), and resulted in a four-fold significant reduction in these parameters. This is shown by the shift of Flocks 10 and 11 back along the first canonical variate into C2 (Fig 1). Therefore the management practice of both the partial and total house clean-out have significant effects on these parameters in the soil underlayment. This finding is vital for the development of future management practices, as it is clear that these clean-outs have the potential to significantly reduce the potential for infiltration of heavy metal pollutants into the ground water and the potential related pollution Table 3.

### Parameter (OM)

**Fig 7:**
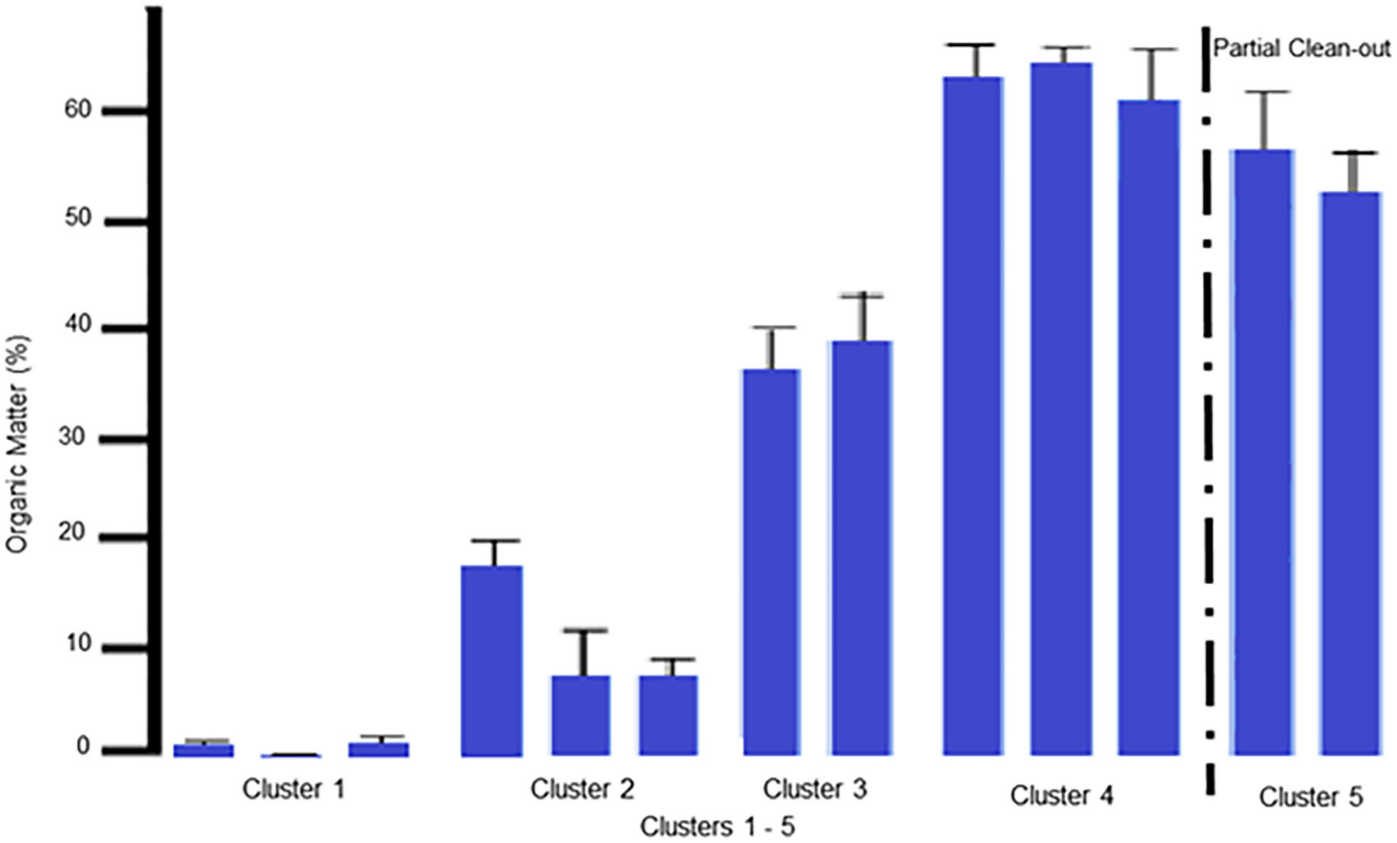
Mean values for Organic Matter. Mean values for Organic Matter, expressed as a percentage, with standard deviation error bars are shown in the bottom graph. In order from left to right on the graph is Cluster 1: Native, Pad and Flock1; Cluster 2: Flock 2, 10 and 11; Cluster 3: Flocks 3 and 4; Cluster 4: Flocks 5, 6, and 7; and Cluster 5: Flocks 8 and 9. The partial clean-out which occurred between Flock 7 and 8 is marked by a dashed line.

The OM displayed a continued increase (Fig 7) with each successive cluster prior to the partial clean-out, after Flock 7, (C1–4), (11-fold, two-fold, and two-fold, respectively). These increases can easily be attributed to the buildup of manure and urine of the successive flocks of birds utilizing the same litter. As the manure and urine increases in the litter, more organic matter infiltrates into the underlying soil thus driving the shift observed. The partial clean-out resulted in a 1.5-fold decrease between C4 and 5; this negative shift of C5 along the second canonical variate is clearly shown in (Fig 1).

## Conclusion

Reuse of litter in the same house for multiple broiler flock grow-outs, without a complete clean-out, has become a common management practice in the poultry industry; due to the rising cost and the difficulties of procuring bedding materials. This study is the first to evaluate and demonstrate that as successive flocks are reared on the same litter the capacity of that litter to absorb potential soil and ground water contaminants is significantly reduced resulting in changes to the underlying soil. These results clearly establish that a partial clean-out has limited impact on maintaining the pre-flock profile of the underlying soil. In contrast, the total clean-out management procedure significantly reduced all parameters of the underlying soil (with the exception of pH) to levels trending lower but not significantly different from those observed in second flock rotation. Therefore when economically and logistically feasible, the incorporation of more frequent total clean-outs into routine management practices could help reduce the risk of infiltration of potential pollutants from the poultry litter into the soil underlayment and possibly the ground water. This work also demonstrates a need to be thoughtful about placement of poultry house clean-out materials. Future research in this arena could also examine the possibility of adding a layer of elemental sulfur after clean-outs; as sulfur is known to decrease pH in alkaline soils [29]
